# Supplementary material for: Velvet Family Members Regulate Pigment Synthesis of the Fruiting Bodies of Auricularia cornea
Source: J Fungi (Basel). 2023 Mar 27;9(4):412. doi: 10.3390/jof9040412 (PMC10140996; doi:10.3390/jof9040412)
Supplement: Supplementary file 1 [file jof-09-00412-s001.zip › Table S2.pdf]

Table S2. Primer of RT-qPCR

Primer number	Left Primer	TM(℃)	Right Primer	TM(℃)
AcveA-1	GGCGCTACCTCGTATGTCTGG	62	CGATAACCTCCCCAGGCAACC	62
AcveA-2	TCAGGCCTTGCCGTTGACAG	62	CGTCGTCCAAGGCATCCGT	62
AcvelB-1	CCGCAACGTGAATGATGCTGAG	61	CGGCCGTGGATCTCTGGTC	61
AcvelB-2	TTACTCGACCCGTGGCGG	62	GCGTTGTCGATTGCGTTCGG	61
