# Supplementary material for: Velvet Family Members Regulate Pigment Synthesis of the Fruiting Bodies of Auricularia cornea
Source: J Fungi (Basel). 2023 Mar 27;9(4):412. doi: 10.3390/jof9040412 (PMC10140996; doi:10.3390/jof9040412)
Supplement: Supplementary file 1 [file jof-09-00412-s001.zip › Table S3.pdf]

Table S3. Monokaryons and the fruiting body color of backcross population

Monokaryotic strain number of mapping population	Backcrossed with ACW001-33	Backcrossed with ACP004-33	Color of fruiting body
1	+	−	P
2	+	−	W
3	+	−	W
4	+	−	W
5	−	+	P
6	+	−	P
7	−	+	P
8	−	+	P
9	−	+	P
10	−	+	P
12	−	+	P
13	−	+	P
14	−	+	P
15	−	+	P
17	+	−	W
18	−	+	P
19	+	−	P
21	+	−	W
22	+	−	W
23	+	−	W
26	−	+	P
27	−	+	P
30	−	+	P
31	−	+	P
32	−	+	P
34	+	−	W
35	−	+	P
36	−	+	P
37	+	−	W
38	−	+	P
39	−	+	P
40	+	−	W
41	−	+	P
42	−	+	P
43	−	+	P
45	−	+	P
46	−	+	P
47	+	−	W
48	−	+	P
49	−	+	P
50	+	−	W
51	−	+	P
52	+	−	W
53	−	+	P

54	+	−	W
55	−	+	P
56	−	+	P
57	−	+	P
58	−	+	P
59	−	+	P
60	−	+	P
61	+	−	P
62	+	−	W
63	+	−	P
65	+	−	P
66	−	+	P
67	−	+	P
68	+	−	W
69	−	+	P
71	−	+	P
72	+	−	W
73	−	+	P
74	−	+	P
76	−	+	P
77	−	+	P
79	−	+	P
80	+	−	W
81	−	+	P
82	−	+	P
83	−	+	P
84	−	+	P
85	−	+	P
86	−	+	P
87	−	+	P
88	+	−	P
89	+	−	W
91	−	+	P
92	−	+	P
93	+	−	W
94	+	−	W
95	+	−	P
96	+	−	W
97	+	−	W
98	−	+	P
99	+	−	W
100	+	−	W
101	−	+	P

* “P” represents the color of the fruiting body is purple. “W” represents the color of the fruiting body is white.
