# Supplementary material for: Velvet Family Members Regulate Pigment Synthesis of the Fruiting Bodies of Auricularia cornea
Source: J Fungi (Basel). 2023 Mar 27;9(4):412. doi: 10.3390/jof9040412 (PMC10140996; doi:10.3390/jof9040412)
Supplement: Supplementary file 1 [file jof-09-00412-s001.zip › Table S4.pdf]

Table S4. Fruiting body color of selfing population of ACW001-33×ACP004-33

A_{w1} \ A_{p1}	3（W）	6（P）	17（W）	22（W）	23（P）	34（W）	40（W）	47（W）	50（W）	54（W）	62（W）	68（W）	72（W）	80（W）	88（P）
5（W）	*	P	W	*	W	W	W	*	W	*	*	*	*	W	P
7（P）	P	P	P	W	W	*	W	W	W	P	P	W	*	W	P
9（W）	W	P	W	W	W	W	W	*	W	W	W	W	W	W	P
14（W）	*	P	W	W	W	*	W	W	W	*	W	W	*	W	P
26（W）	*	P	*	*	W	*	*	W	W	W	*	W	*	W	*
38（W）	W	P	W	W	W	W	W	W	W	W	W	W	*	*	P
45（W）	W	*	*	*	*	*	W	*	*	W	*	W	*	W	*
48（P）	*	P	P	P	P	P	P	P	P	P	P	*	*	P	*
51（P）	P	P	P	*	W	W	W	W	*	P	P	W	*	W	P
56（P）	*	P	P	P	P	P	*	P	P	*	*	P	*	*	P
71（W）	W	*	W	*	W	W	*	W	W	*	W	W	W	*	P
76（W）	W	P	W	*	W	*	*	*	W	*	*	W	*	W	*
86（W）	*	*	W	W	P	W	W	W	W	*	W	W	W	W	P
91（W）	W	P	*	W	*	W	*	*	W	W	*	W	*	W	P

* “P” represents the color of the fruiting body is purple. “W” represents the color of the fruiting body is white. “ * ” represents cross was successful but didn’t produce fruiting body.
