# Supplementary material for: Velvet Family Members Regulate Pigment Synthesis of the Fruiting Bodies of Auricularia cornea
Source: J Fungi (Basel). 2023 Mar 27;9(4):412. doi: 10.3390/jof9040412 (PMC10140996; doi:10.3390/jof9040412)
Supplement: Supplementary file 1 [file jof-09-00412-s001.zip › Table S5.pdf]

Table S5. Fruiting body color of selfing strains and the types of corresponding monokaryotic strains

Monokaryon of mapping population	Mated with 23	Mated with 51	Color of fruiting body	Monokaryon type
1	−	+	P	P_d
2	−	+	W	P_r
3	−	+	P	W_r
4	−	+	W	P_r
5	+	−	W	W_d
6	−	+	P	P_d
7	+	−	W	P_r
8	+	−	*	*
9	+	−	W	W_d
10	+	−	W	W_d
12	+	−	*	*
13	+	−	W	W_d
14	+	−	W	W_d
15	+	−	*	*
17	−	+	P	W_r
18	+	−	P	P_d
19	−	+	P	P_d
21	−	+	W	W_d
22	−	+	*	*
23	−	+	W	P_r
26	+	−	W	W_d
27	+	−	P	W_r
30	+	−	P	W_r
31	+	−	P	P_d
32	+	−	P	W_r
34	−	+	W	W_d
35	+	−	W	W_d
36	+	−	*	*
37	−	+	P	W_r
38	+	−	W	W_d
39	+	−	P	W_r
40	−	+	W	W_d
41	+	−	P	W_r
42	+	−	*	*
43	+	−	*	*
45	+	−	*	*
46	+	−	*	*
47	−	+	W	W_d
48	+	−	P	P_d
49	+	−	*	*
50	−	+	*	*
51	+	−	W	P_r
52	−	+	W	W_d
53	+	−	*	*
54	−	+	P	W_r

55	+	−	P	P_d
56	+	−	P	P_d
57	+	−	W	W_d
58	+	−	⁎	⁎
59	+	−	P	W_r
60	+	−	W	W_d
61	−	+	⁎	⁎
62	−	+	P	W_r
63	−	+	⁎	⁎
65	−	+	P	P_d
66	+	−	P	P_d
67	+	−	W	P_r
68	−	+	W	W_d
69	+	−	W	W_d
71	+	−	W	W_d
72	−	+	⁎	⁎
73	+	−	P	P_d
74	+	−	⁎	⁎
76	+	−	W	W_d
77	+	−	⁎	⁎
79	+	−	⁎	⁎
80	−	+	W	W_d
81	+	−	⁎	⁎
82	+	−	W	W_d
83	+	−	P	P_d
84	+	−	W	P_r
85	+	−	P	W_r
86	+	−	P	W_r
87	+	−	⁎	⁎
88	−	+	P	P_d
89	−	+	⁎	⁎
91	+	−	⁎	⁎
92	+	−	P	W_r
93	−	+	⁎	⁎
94	−	+	W	W_d
95	−	+	P	P_d
96	−	+	⁎	⁎
97	−	+	P	W_r
98	+	−	W	W_d
99	−	+	P	W_r
100	−	+	W	W_d
101	+	−	P	W_r

* "P" represents the color of the fruiting body is purple. "W" represents the color of the fruiting body is white. " ⁎ " represents cross was successful but didn't produce fruiting body.
