# Supplementary material for: Velvet Family Members Regulate Pigment Synthesis of the Fruiting Bodies of Auricularia cornea
Source: J Fungi (Basel). 2023 Mar 27;9(4):412. doi: 10.3390/jof9040412 (PMC10140996; doi:10.3390/jof9040412)
Supplement: Supplementary file 1 [file jof-09-00412-s001.zip › Table S6.pdf]

Table S6. The sequence of SSR primers

Primer number	Left Primer	TM(℃)	Right Primer	TM(℃)
SSR-1	ccattgatttattcgaccaaagtt	60.443	gtgtgttgggtttgtgggac	61.134
SSR-4	gcattctcagcatcgaagacata	61.626	ctgagcagcagcctcctctt	62.314
SSR-6	agcgagttcgacgtgtcttc	60.599	gtcccaactttggttggctt	61.275
SSR-8	aactctctgggaattcatatggc	60.679	ctctcagcgatgaaagctcac	60.292
SSR-12	attaccctgaacactgtcattgg	60.158	ctgccttgagtcatgtggttagt	60.594
SSR-462	gagtgaacgcttattcaccatct	59.672	cgggggatataatttatctaggg	59.052
SSR-464	gttatagccttcgagtcggattt	60.007	agaacctagaggctctttccgt	59.915
SSR-466	gtaacatgaagtccgccacc	60.384	gatgaactacacataccaaggctg	59.954
SSR-470	cttgagatctcgaacctgaaacg	62.472	gggaggatgaaggagactaaacc	61.495
SSR-476	ggtctggggtgtacgtcgag	62.89	cacagctcgaaatcccacaa	62.175
SSR-959	ggtctggggtgtacgtcgag	62.89	cacagctcgaaatcccacaa	62.175
SSR-960	taatgcttctgtcttcctttccc	60.924	cgtgatctacatgctgtcaaaga	60.319
SSR-965	gagactcgaacagcggacaag	62.447	cccactcaccgactcctctc	62.223
SSR-966	ttttagaggggatgtgaggtagc	60.82	ctgagcaggctcttcccttac	60.522
SSR-1321	gtcttcgagcgtgtgtagatga	60.462	ctcgttggattccagcctac	59.694
SSR-1326	gttccaggttgcgtaaaatga	59.989	ccaagcgtcaaagataggtaaaa	59.687
SSR-1328	gtgccgttaatctttctcatcc	59.972	atacgagtgtatgatccacaggg	60.144
SSR-1329	tacgctgacgaaaagtccga	61.869	cacatgcatctggacacaagag	61.712
SSR-1331	cagaaagcccttcaacctagaat	60.134	gagtctgataccgactcttcgc	60.411
SSR-1337	gtagagctggaattggctgtg	59.89	atcaagcgtcatcatgtcaaaa	60.511
SSR-1341	agctgctgtactctcagtcgg	59.824	cactccatctccatcgacac	59.05
SSR-1734	ttatgtatatagctccgaagcgg	59.67	caactgatagatcccgctatgac	60
SSR-1742	ggtttatttggtcgttgtttgtt	59.236	aatagcattgcgatactgaaacag	59.725
SSR-1745	gacataatactgtgcagtgtcgg	59.576	gctgctccattgagaaagagat	59.99
SSR-1752	ctacagccacttcaacagcatac	58.988	ccctggatttattgagacacatc	59.717
SSR-1759	caacccgacccttgactctc	62.009	gaggcgagtactcctcatcagc	62.696
SSR-1769	ttgcgccatgatatcgtagtagt	60.875	aggatgtatgctgggtccaat	60.593
SSR-2165	aaccaaacctaacctaacccaac	59.591	tgcaccaaaaacactgattagc	60.173
SSR-2171	ttagggtactgctcctcaaactg	59.827	gcaaacttcgcaaggagatt	59.457
SSR-2179	actcatgtttgtccttgctgaat	60.054	gttgccctgtactcaaggtacg	60.95
SSR-2183	caagaagattatcatgacgcaga	59.375	tcaagtactgagagaaaaacgcc	59.941
SSR-2193	cagatgacccattctgcgta	59.673	cactcgctcgagaaatacctg	60.023

SSR-2206	agcctttacgccacaacctac	60.547	tgctgagatacttgtgcagactc	59.699
SSR-2474	aaggtcgaagttaccagggc	60.496	taatcctcctggggatcgag	61.307
SSR-2482	acagcaacggtatctcatctcat	60.04	gaacggaaacgaaaaataaggat	59.755
SSR-2483	gagttgaggggtggtcctct	60.504	actgtctacgctttacaaatggttc	60.008
SSR-2487	ccacacgtactaccacatcatcc	61.465	attttgtcccagagcgtggt	61.84
SSR-2491	gaagacacgtatacgcacattca	60.074	aacgcgtattactgcatcaactt	60.095
SSR-2499	actacggtgagtcgacagcg	61.469	gtgcagttctgaacgaaagagtc	60.47
SSR-2503	caacaacgtcgtctataattggg	60.63	tgataatgttcgcgtacgactg	61.065
SSR-2507	gcctcaaatatcgtggtgttatc	59.756	cggataattgtctctggtttctg	60.008
SSR-2511	gatagcgacagtgacacagacag	59.991	gtagagcgagcgtttgacctt	60.937
SSR-2836	accactatgccagtgttcgag	60.186	agactgctgaagagagtggtcag	60.242
SSR-2848	aagggagataccaggtgtgtttt	60.155	aaagggtacatcactcacaggaa	59.914
SSR-2863	gatgcgagaggcataggatg	60.733	aagaagctcactgctgaggtg	59.801
SSR-3096	gaggtccatcctgttcatgc	60.484	ctcactcactcgccgatagc	61.107
SSR-3098	aacagtgtatgcttgcgtatgtg	60.13	tcttctctggctgtgatctctct	59.776
SSR-3104	attccacaggaatttcatggc	61.068	gcacacgaggtaaccatcttaca	61.311
SSR-3122	ctgatgtacactctgctgctcac	60.133	ggaacagcgatatcttcctgata	59.606
SSR-3124	aagaaacacattttgcgctgtat	60.076	ttgtagtgacggcactcgtatta	59.72
SSR-3137	gtctgatttcgcaacgacag	59.445	attatagaacatcctcgccgtta	59.082
SSR-3279	gactacttctaccgcactccaaa	59.833	cttcacgagtgggacaaagtc	59.754
SSR-3290	gatactacgattgcagtcagcg	59.933	aaggcgagagtctccaactatg	59.907
SSR-3297	agggaacaaaagggaaataaggt	60.426	atgctgtttccacagaaaaacac	60.438
