# Supplementary material for: Velvet Family Members Regulate Pigment Synthesis of the Fruiting Bodies of Auricularia cornea
Source: J Fungi (Basel). 2023 Mar 27;9(4):412. doi: 10.3390/jof9040412 (PMC10140996; doi:10.3390/jof9040412)
Supplement: Supplementary file 1 [file jof-09-00412-s001.zip › Table S7.pdf]

Table S7. PTG series fine mapping primers

Primer number	Left Primer	TM(℃)	Right Primer	TM(℃)
PTG-1	gctctccatgaatgggtgaagct	61	caccccttccagaagtcggg	60
PTG-2	tcgtcatcgtccctgagcaac	61	taggtgcagacccctctctgt	61
PTG-4	cggcaccgacttcggaaag	60	ttgcgcaaaggctgcttctc	61
PTG-10	ggccgcagcagctcaaca	62	cacgctgcaaggaacggt	60
PTG-11	gttgtcgccagcttcgct	60	cgacatcgtcgtgccagc	61
PTG-20	ggatatacccgctctcgcaatcc	60	ccgttatggcacaagccagc	60
PTG-21	agacctcgttaattgacggcaac	60	cgtcgaggtcgcagtatcaagc	61
